# Implications of isonicotinylation-associated patterns in NK cells in the pathogenesis of Kawasaki disease: evidence from artificial intelligence-driven multi-omics and clinical validation

**DOI:** 10.3389/fped.2026.1870229

**Published:** 2026-07-22

**Authors:** Yanli Yang, Pengjuan Hu

**Affiliations:** Department of Cardiovascular and Rheumatology Immunology, Children’s Hospital of Shanxi Province, Taiyuan City, China

**Keywords:** artificial intelligence, isonicotinylation, Kawasaki disease, multi-omics, NK cells

## Abstract

**Background:**

Kawasaki disease (KD) is a systemic vasculitis of childhood driven by aberrant immune activation. Natural killer (NK) cell dysregulation plays a critical role, but its upstream molecular mechanisms remain unclear. Isonicotinylation (Kinic), a novel lysine acylation acting as a metabolic sensor, represents an unexplored regulatory layer in KD.

**Methods:**

We employed an artificial intelligence (AI)-driven multi-omics framework. Limma differential expression analysis on GSE68004 (KD patient bulk profile) identified Kinic-related differentially expressed genes. Weighted gene co-expression network analysis (WGCNA) and CIBERSORT on GSE18606 (KD patient bulk profile) delineated an NK cell-correlated module. Their intersection defined Kinic- and NK (KN)-related signature, which was used to construct a diagnostic model via an explainable machine learning pipeline on KD patient bulk profiles (GSE73463, GSE73462, and GSE63881). The central hub gene was validated using KD patient single-cell RNA-sequencing (scRNA-seq) data (GSE24757). An AI-based drug screen (DrugReflector) and molecular docking nominated therapeutic candidates. Finally, PPID expression was validated in an independent clinical cohort using q-RT-PCR.

**Results:**

We identified a six-gene KN-associated signature that demonstrated excellent diagnostic performance. PPID can be considered upregulated hub gene. Single-cell analysis confirmed predominant PPID expression in NK cells and linked its function to epigenetic modification and inflammatory signaling. Drug screening nominated BRD-K90382497 as a potential PPID-targeting compound.

**Conclusion:**

This study unveils a novel KN-associated molecular axis in KD pathogenesis, with PPID as an NK cell-centric hub. This axis provides a promising diagnostic biomarker and identifies a potential therapeutic target, bridging a novel metabolic modification to NK cell dysfunction in KD.

## 1 Introduction

Kawasaki disease (KD) is an acute, self-limited vasculitis of unknown etiology that predominantly affects children under 5 years of age, representing the leading cause of acquired heart disease in developed nations (1). The pathogenesis of KD is complex and incompletely understood, involving a robust and dysregulated innate and adaptive immune response triggered by an unknown infectious or environmental agent in genetically susceptible individuals (2).

Among the immune cells implicated in KD, natural killer (NK) cells have gained increasing attention. NK cells are innate lymphoid cells that play a crucial role in the early immune response against viral infections and in tumor surveillance (3). In the acute phase of KD, NK cells are activated, exhibit altered cytotoxicity, and produce high levels of pro-inflammatory cytokines such as IFN-γ, which contribute to endothelial activation and vascular damage (4). Beyond the classical involvement of cytokines and immune cells, emerging evidence highlights the role of post-translational modifications (PTMs) in modulating protein function and KD progression (5). Isonicotinylation (Kinic) is a recently characterized lysine acylation, in which an isonicotinoyl group is transferred onto lysine residues, potentially altering protein stability, interactions, and activity (6). This modification, linked to cellular metabolic states, has been implicated in cancer and certain inflammatory conditions but remains entirely uninvestigated in the context of KD or NK cell biology (6).

To address this gap, we conducted an integrative multi-omics study leveraging artificial intelligence (AI) to delineate the coordinated role of Kinic–NK cell biology in KD. We aimed to identify a shared isonicotinylation–NK (KN)-associated molecular signature in KD patients with corresponding diagnostic and patient-stratifying potential, as well as potential therapeutic availability, focusing on the identification of a central hub gene using explainable AI.

## 2 Material and methods

### 2.1 Source of bulk data

Bulk RNA-sequencing datasets from peripheral blood of KD patients and healthy controls (HC) were retrieved from the GEO database. The datasets were strategically assigned. GSE68004 served as the internal set 1 (comprising 37 HC samples, 76 KD samples), GSE18606 served as the internal set 2 (comprising 9 HC samples, 39 KD samples), integrated GSE73463 and GSE73462 (comprising 16 HC samples, 359 KD samples) were considered the training set, and GSE63881 served as the independent validation set (comprising 117 HC samples and 171 KD samples). Batch effects were assessed and corrected using the sva R package (7, 8). A curated Kinic-associated gene signature was compiled from relevant literature (9).

### 2.2 Limma analysis

Differential expression analysis between KD and HC groups in GSE68004 was performed using the limma R package (|log2FC| > 0.5, *p* < 0.05) (7). The intersection with the Kinic-related gene set yielded Kinic-related differentially expressed genes (DEGs), which were analyzed by KEGG and GO enrichment analysis using the clusterProfiler package in R, referencing KEGG and GO gene sets downloaded from the MSIGDB database (10). Kinic-related DEG intersected patterns were assessed by STRING database and Cytoscape software.

### 2.3 CIBERSORT and WGCNA analysis

The relative abundance of NK cells in GSE18606 was estimated using CIBERSORT package in R (11). Weighted gene co-expression network analysis (WGCNA) was performed using the WGCNA R package to construct a scale-free co-expression network (12). The module showing the highest absolute correlation with the NK cell infiltration score was selected as the NK cell-correlated module (12). Genes from this module were intersected with Kinic-related DEGs to obtain KN-associated DEGs.

### 2.4 Systemic machine learning model construction

A comprehensive machine learning workflow was implemented using caret and glmnet on the training set (GSE73463 and GSE73462) (13). The 132-model machine learning framework typically represents a comprehensive grid search approach that systematically combines multiple classification algorithms with various feature selection methods and preprocessing strategies. The combinations of these algorithms were applied to the MetaGSE for signature selection and model construction using 10-fold cross-validation (13). The optimal model [RF + Enet(alpha = 0.2)] was selected based on the highest mean area under the receiver operating characteristic curve (AUC-ROC) (13). Its performance was evaluated on the independent validation set (GSE63881) (13). A nomogram coupled with calibration was constructed via rms package in R (13). Shapley additive explanations (SHAP) analysis was applied to identify the hub gene with the greatest contribution (9).

### 2.5 Single-cell analysis

scRNA-seq data (GSE24757, including two KD patient peripheral blood samples) were acquired from the GEO database and processed using the Seurat pipeline in R (14). Cells with unique feature counts <200 or >6,000, or with >15% mitochondrial gene content were filtered out (14). Data were normalized, and the top 2,000 highly variable genes were identified (14). Principal component analysis (PCA) was followed by graph-based clustering and visualization with uniform manifold approximation and projection (UMAP) and t-distributed stochastic neighbor embedding (t-SNE) (14). Cell types were annotated using canonical markers through the SingleR package in R (15). The expression distribution of the hub gene was visualized across all cell types. Cell communication patterns were assessed by CellphoneDB package in R (16). Pseudotime trajectory analysis of NK cells was conducted using Monocle2 package in R (17). A virtual knockout of hub gene in the NK cell cluster was simulated using scTenifoldKnk package in R, and downstream functional impact was assessed by KEGG and GO enrichment analysis, referencing KEGG and GO gene sets downloaded from the MSIGDB database (18). AUCell was performed to identify Kinic patterns across cell types in reference to KEGG and GO gene sets downloaded from the MSIGDB database (19).

### 2.6 Drug screening

DrugReflector was employed to screen the cMAP database for compounds predicted to reverse the KD gene expression signature to HC in integrated GSE73463 and GSE73474 (20). The top candidate compound was selected (20). Molecular docking between the PPID protein and the candidate drug was performed using AutoDock Vina software and visualized with PyMOL software (21).

### 2.7 Clinical sample estimation

To validate the translational relevance of our computational findings, we examined hub gene LTB expression in peripheral blood samples from an independent clinical cohort. Fresh blood (8–10 mL per donor) was collected in ethylenediaminetetraacetic acid (EDTA) tubes from six patients with KD and six age- and sex-matched HCs. Peripheral blood mononuclear cells (PBMCs) were isolated by density gradient centrifugation. In brief, blood was diluted 1:1 with phosphate-buffered saline (PBS) (Gibco, Grand Island, NY, USA), layered over an equal volume of Ficoll-Paque PLUS (Cytiva, Marlborough, MA, USA), and centrifuged at 400 × g for 30 min at room temperature with the brake off. The PBMC layer was carefully collected, washed twice with PBS (300 × *g*, 10 min), and either processed immediately for RNA extraction or stored in TRIzol reagent (Invitrogen, Carlsbad, CA, USA) at −80 °C. Total RNA was extracted using TRIzol™ Reagent (Invitrogen) following the manufacturer's protocol, and RNA concentration and purity were evaluated with a NanoDrop™ One spectrophotometer (Thermo Fisher, Waltham, MA, USA). Then, 1 µg of total RNA was reverse-transcribed into cDNA using the PrimeScript™ RT reagent Kit with gDNA Eraser (Takara Bio, Kusatsu, Shiga, Japan) to remove genomic DNA. Quantitative PCR was performed on a QuantStudio™ 5 Real-Time PCR System (Applied Biosystems, Foster City, CA, USA) using TB Green® Premix Ex Taq™ II (Tli RNaseH Plus) (Takara Bio, Japan). The primer sequences for PPID and the GAPDH were as follows.

PPID:

F: 5′-AATCAGAATGGGACAGG-3′

R: 5′-AACGCACAATTTAGCAG-3′

GAPDH:

F: 5′-GACCTGACCTGCCGTCTAG-3′

R: 5′-AGGAGTGGGTGTCGCTGT-3′

### 2.8 Statistical analysis

Statistical analyses were performed in R (version 4.2.2) or GraphPad Prism (version 9.0). For two-group comparisons, Student's *t*-test or Mann–Whitney *U* test was applied as appropriate. For multiple group comparisons, one-way ANOVA with Tukey's *post-hoc* test was used. A *p*-value <0.05 was considered statistically significant.

## 3 Results

### 3.1 Identification of Kinic-associated DEGs for KD patients

**Figure 1:**
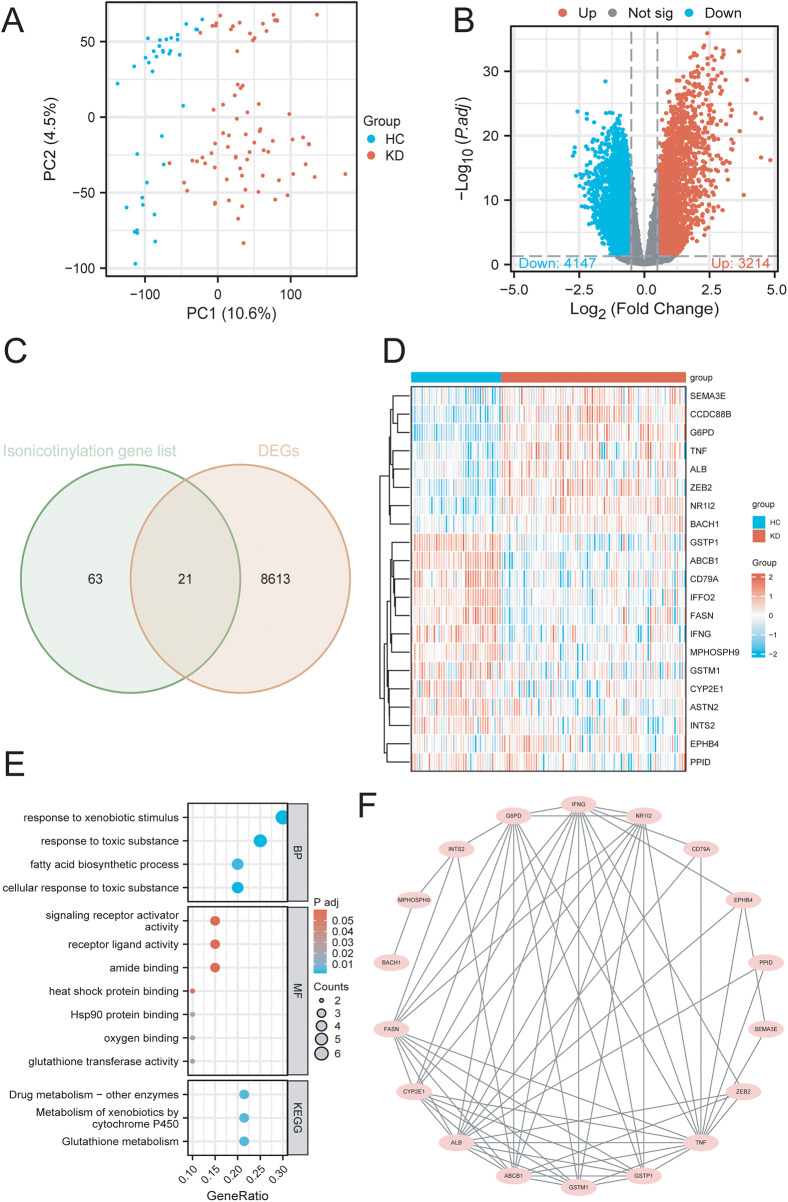
Identification of Kinic-related DEGs in KD. **(A)** PCA plot illustration of results of removal of batch effects in GSE68004. **(B)** Volcano plot displaying DEGs in GSE68004 (KD group compared to HC group). **(C)** Venn diagram illustrating the intersection between all DEGs and the Kinic-related gene set, yielding 21 core Kinic-associated genes. **(D)** Heatmap depicting the expression patterns of the 21 Kinic-related DEGs in KD bulk profile (GSE68004). **(E)** KEGG and GO enrichment analysis of the molecular functions of 21 Kinic-related DEGs. **(F)** STRING database enrichment analysis of 21 Kinic-related DEGs molecular interaction patterns.

Differential expression analysis of GSE68004 identified a total of 7,361 DEGs between KD and HC (|log2FC| > 0.5, *p* < 0.05), including 3,214 upregulated DEGs and 4,147 downregulated DEGs after elimination of batch effects (Figures 1A,B). Intersection with the Kinic-related gene set yielded 21 significant Kinic-related DEGs (Figure 1C). Expression patterns between KD and HC were assessed by 21 significant Kinic-related DEGs (Figure 1D). Functional enrichment analysis showed these genes were enriched in processes related to epigenetic modification, such as acetylation and inflammatory signaling (Figure 1E). Next, these 21 significant Kinic-related DEGs interacted patterns were assessed to gain insights into their molecular interaction patterns (Figure 1F).

### 3.2 Identification of NK cell-associated DEGs for KD patients

**Figure 2:**
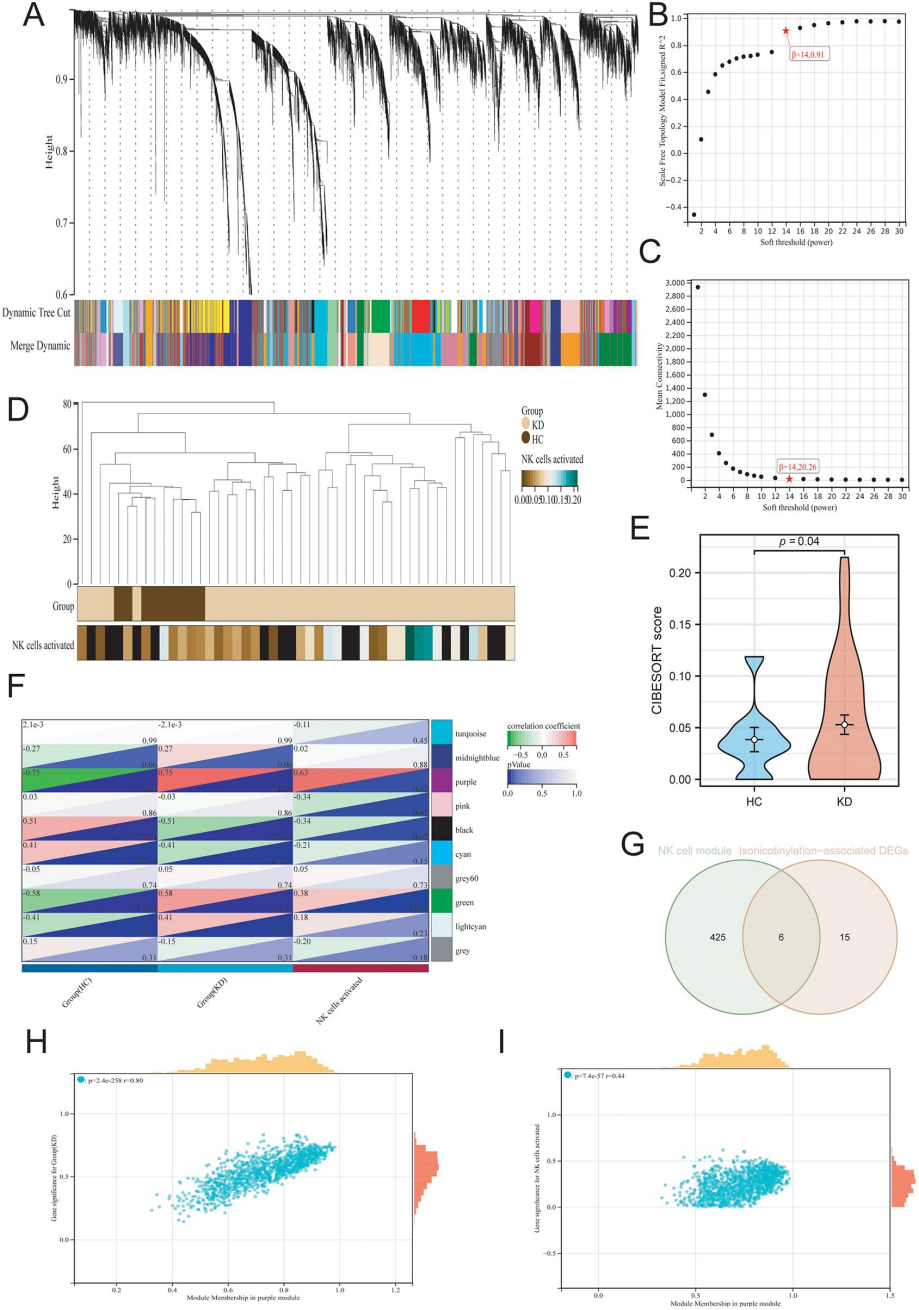
WGCNA identifies an NK-correlated module and defines the KN signature in GSE18606. **(A)** Cluster dendrogram of genes from GSE18606, with colors indicating assigned modules. **(B,C)** Analysis of scale-free topology and mean connectivity for selecting the soft-thresholding power (*β*). **(D)** Dendrogram tree of genes from GSE18606, with colors indicating assigned modules. **(E)** Box plot comparing the CIBERSORT-estimated NK infiltration score between KD and HC groups in GSE180606. **(F)** Heatmap of module–trait relationships. **(G)** Venn diagram of six-gene KN-associated signature. **(H,I)** Scatter plot of gene significance for NK infiltration and KD.

CIBERSORT analysis of GSE18606 confirmed a significant alteration in NK cell infiltration in KD samples compared to HC (Figure 2E). WGCNA identified a specific module (purple) that showed the strongest positive correlation with the NK cell infiltration score (Figures 2A–D). This module contained 431 genes (Figure 2F). Intersecting these with the Kinic-related DEGs identified six overlapping genes (Figures 2G–I).

### 3.3 Identification of KN-associated DEGs for KD patients

**Figure 3:**
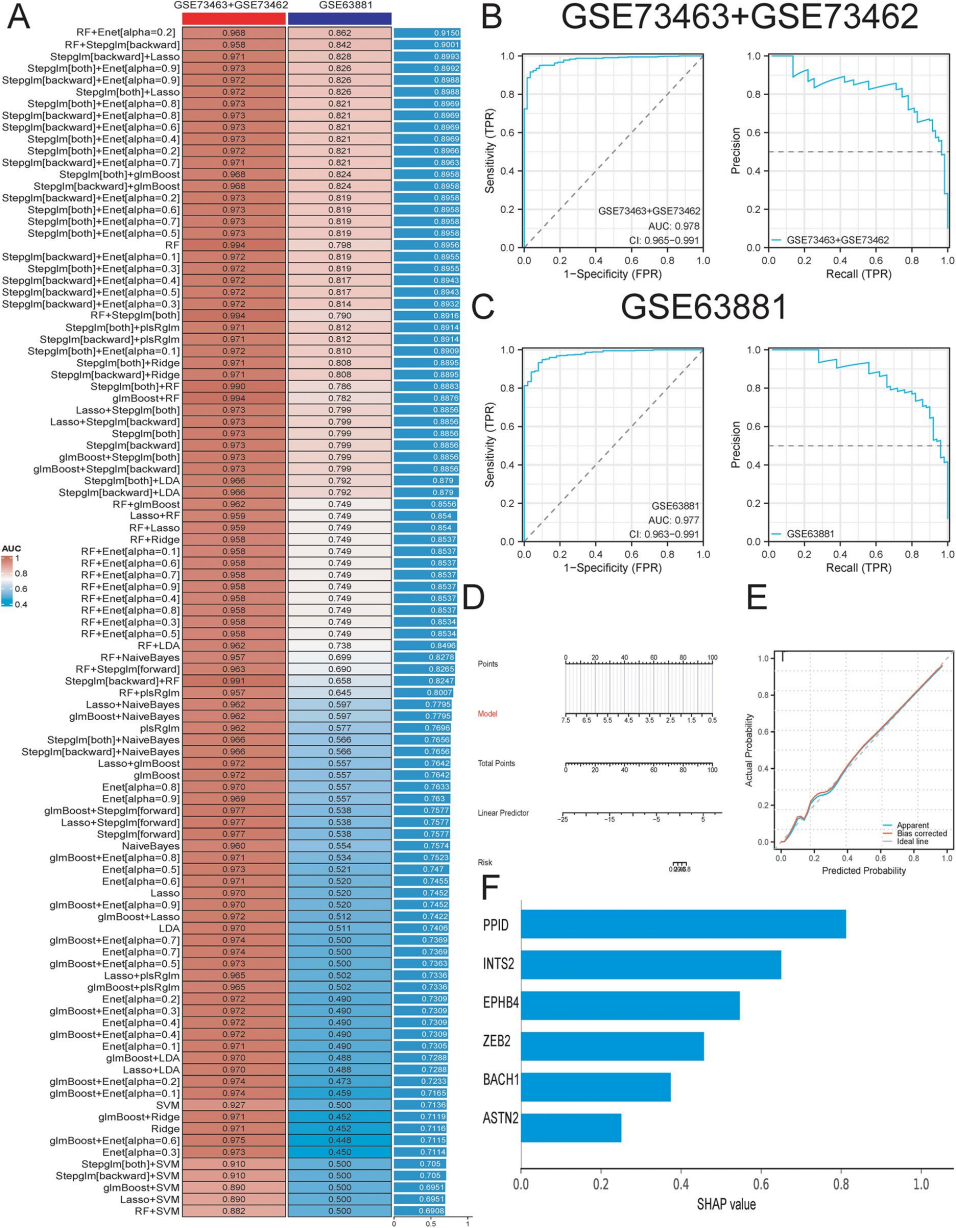
Development and validation of a KN signature-based diagnostic model for KD. **(A)** Performance metrics of 132 machine learning algorithm combinations during 10-fold cross-validation on the training set (GSE73463 and GSE73642). **(B)** ROC and PR curves of the optimal model on the training set. **(C)** ROC and PR curves of the optimal model on the validation set. **(D,E)** Nomogram and calibration plots. **(F)** SHAP summary plot, ranking the contribution of each KN-related signature gene to the model output.

In the training set (GSE73463 and GSE73462) and validation set (GSE63881), 132 machine learning model combinations were evaluated (Figure 3A). The RF + Enet(alpha = 0.2) model achieved an AUC of 0.978 in training set and AUC of 0.977 in validation set (Figures 3B,C). Model efficacy was examined via nomogram and calibration analysis in the training set, demonstrating favorable diagnostic efficacy (Figures 3D,E). SHAP summary plots unequivocally identified PPID as the most influential feature driving the model predictive power, establishing it as the central hub of the KN-associated axis (Figure 3F).

### 3.4 Single-cell atlas of NK cell in KD patients

**Figure 4:**
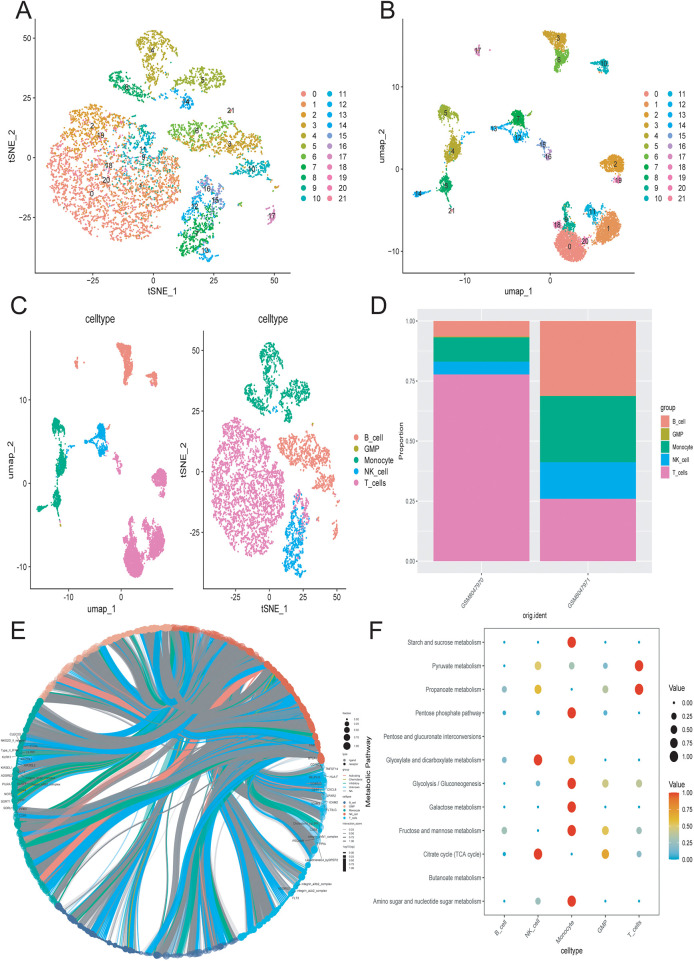
Single-cell characterization of the immune microenvironment in KD. **(A,B)** UMAP and t-SNE of cell clusters. **(C)** UMAP and t-SNE of cell types. **(D)** Bar chart showing the proportion of each cell type across all samples. **(E)** Circle plot of cell–cell communication networks inferred by CellPhoneDB. **(F)** Heatmap of metabolic pathway activity scores across different cell types, analyzed by scMetabolism.

**Figure 5:**
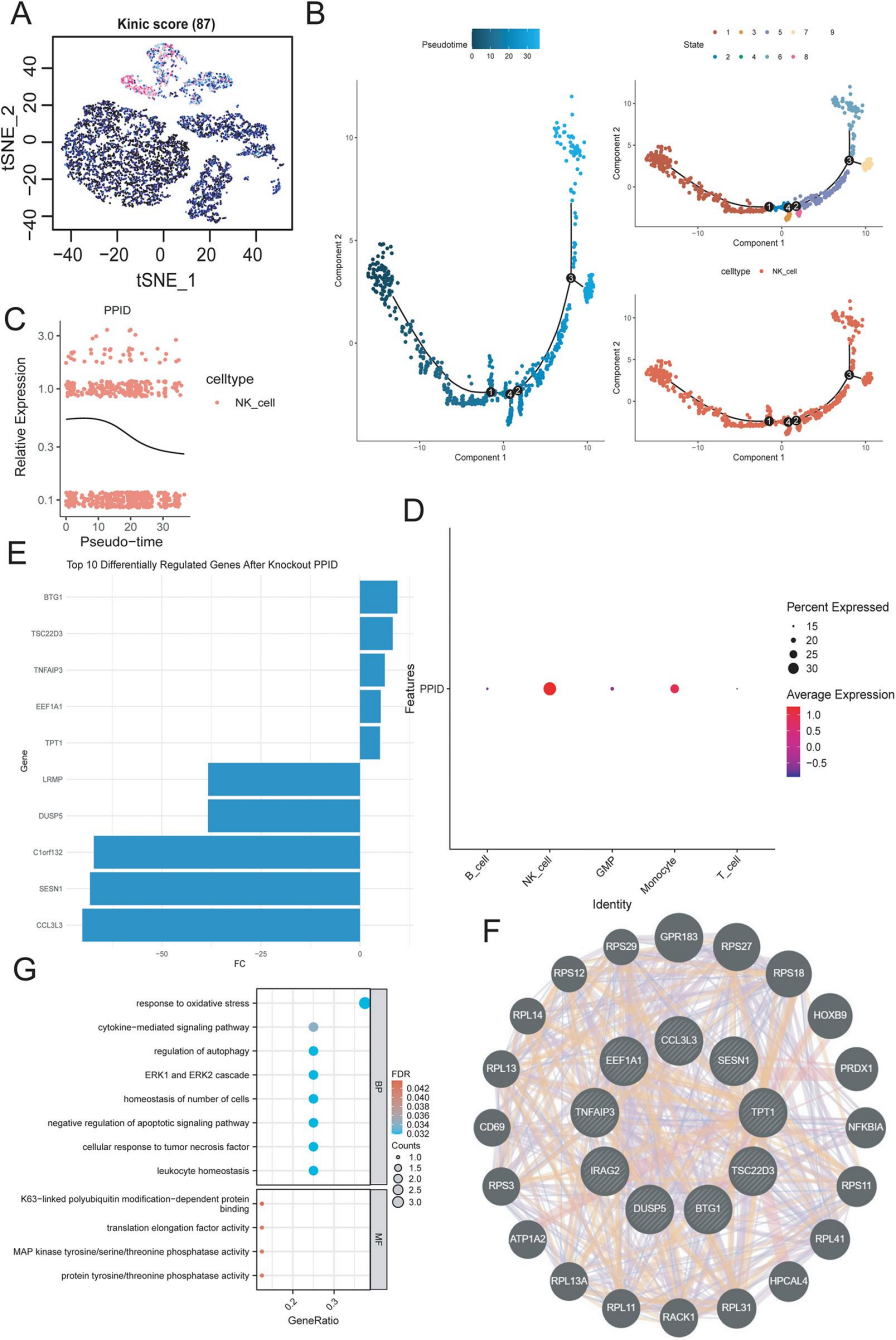
Functional characterization of the KN axis in NK of KD patients. **(A)** AUCell scores for the Kinic gene set across major cell types. **(B,C)** Plot showing the expression dynamics of PPID along the NK cells pseudotime trajectory. **(D)** Distribution of PPID along NK cells. **(E–G)** Results of *in silico* knockout of PPID in NK cells.

Analysis of scRNA-seq data (GSE24757) identified 21 major cell clusters, annotated as five cell types (Figures 4A–C). NK cells and T cells shared a prominent proportion (Figure 5D). Cell–cell communication analysis suggested strong interactions between NK cells and monocytes (Figure 4E). Importantly, metabolic heterogeneity revealed the metabolic pathways in NK cells (Figure 4F).

### 3.5 KN-related signature in NK cells of KD patients

**Figure 6:**
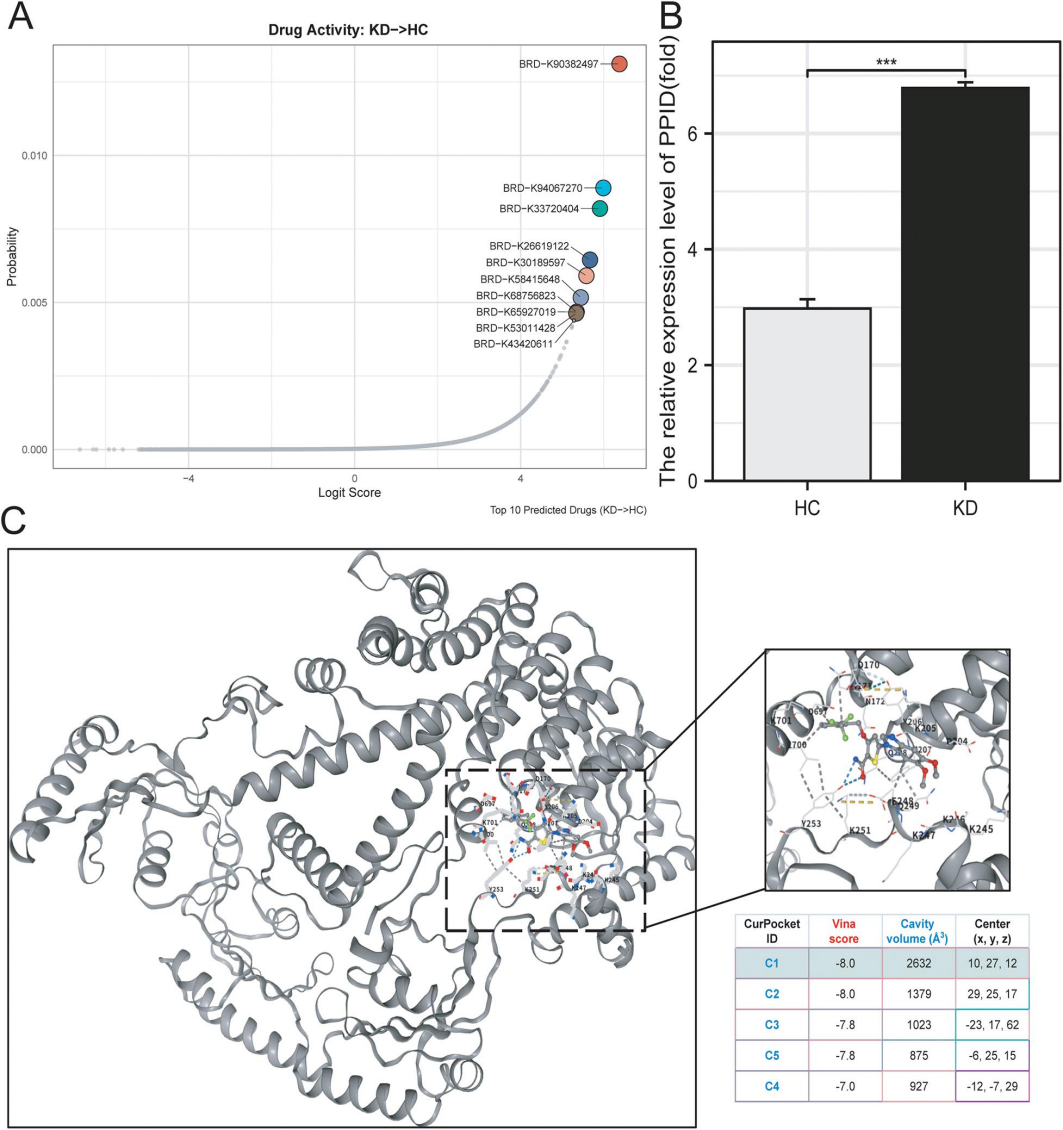
AI-driven drug screening and clinical validation. **(A)** Workflow and result of the DrugReflector screen, nominating BRD-K90382497 as the top potential therapeutic candidate compound predicted to reverse the KD gene signature. **(B)** q-RT-PCR validation showing significantly elevated PPID mRNA levels in PBMCs from KD patients (*n* = 6) compared to HC (*n* = 6). Data are presented as mean ± SEM; ^***^p < 0.001. **(C)** Molecular docking model.

AUCell analysis revealed high activation of the Kinic pathway specifically in NK cells (Figure 6A). Expression analysis at single-cell resolution confirmed that PPID expression was predominantly and specifically localized to the NK cell cluster, with minimal expression in other cell types (Figure 6D). Pseudotime trajectory analysis of NK cells revealed eight differentiation continuum states (Figures 6B,C). *In silico* knockout of PPID in NK cells perturbed a gene network significantly enriched in functions related to epigenetic modification and inflammation (Figures 6E–G).

### 3.6 Drug screening and clinical validation of KN-related signature in KD patients

The DrugReflector screen of the KD signature against the cMAP database nominated BRD-K90382497 as the top potential therapeutic candidate compound predicted to reverse the disease-associated gene expression profile (Figure 6A). Molecular docking simulations predicted a stable and favorable binding interaction between BRD-K90382497 and PPID, with a binding energy of −8.0 kcal/mol, suggesting high affinity (Figure 6C). Finally, clinical sample estimation using q-RT-PCR demonstrated a significant increase in PPID expression in PBMCs from KD patients compared to HC (Figure 6B).

## 4 Discussion and conclusion

This study systematically deciphers a novel molecular axis linking Kinic metabolism to NK cell biology in KD. By employing a rigorous AI-driven multi-omics framework, we identified a six-gene KN-associated signature and established PPID as its NK cell-specific central pathogenic factor. Our findings not only provide a high-performing diagnostic model and a novel patient stratification strategy but also nominate a potential therapeutic compound, thereby bridging a previously uncharacterized post-translational modification to NK cell dysfunction in KD.

PPID, also known as Cyclophilin D or Cyclophilin 40, is a mitochondrial matrix peptidylprolyl isomerase best known for its regulatory role in the mitochondrial permeability transition pore (mPTP), a critical mediator of cell death. Beyond its canonical role in necrosis and apoptosis, emerging evidence has linked cyclophilins to inflammatory signaling and immune cell activation (22). Our study is the first to directly associate PPID with a dysregulated K_inic_ signature in an immune-mediated disease, localize its predominant expression to NK cells at the single-cell level, and demonstrate its potential regulatory role in NK cell function. The *in silico* knockout of PPID in NK cells perturbed a gene network significantly enriched in functions related to epigenetic modification and inflammatory signaling, suggesting that PPID may serve as a crucial metabolic checkpoint in these cells. Besides, PPID was identified as a Kinic-associated modification protein in liver cancer cells (23).

In conclusion, we delineated a novel KN-associated molecular axis in KD pathogenesis through an AI-driven multi-omics approach. The six-gene KN-associated signature serves as an effective diagnostic tool and reveals patient heterogeneity. PPID is established as its NK cell-centric hub, linking metabolic modification to NK cell dysregulation. The nomination of a PPID-targeting compound offers a new translational avenue. These findings illuminate a previously unrecognized immunometabolic pathway in KD, providing fresh insights for biomarker development and targeted therapeutic strategies. However, this study has limitations. The primary analyses rely on publicly available transcriptomic data, which may harbor batch effects or cohort-specific biases. The diagnostic model and subtypes necessitate validation in large, multi-center cohorts with detailed clinical phenotyping for examination of reliability and authenticity of our diagnostic model. The role of Kinic in KD pathogenesis should be further investigated in preclinical studies. In addition, the proposed mechanistic link between Kinic and PPID function remains computational and requires direct biochemical and functional validation through *in vitro* and *in vivo* models. However, the relatively small size of bulk and single-cell data used in our study restrains the reliability of PPID mechanisms in NK cells. Future studies should collect KD patient samples and construct KD animal models to examine PPID molecular and immune patterns in NK for KD pathogenesis. Furthermore, the limited number of patient samples used in our study for examination of PPID expression hinders generalizability. Future studies should include multi-center, large cohorts to examine the pathogenic role of PPID in KD. Finally, the efficacy and safety of the predicted therapeutic compound must be rigorously evaluated in preclinical animal models of KD.

## Data Availability

The datasets presented in this study can be found in online repositories. The names of the repository/repositories and accession number(s) can be found in the article/Supplementary Material.
